# Substance Use, Demographic and Socioeconomic Factors Are Independently Associated With Postpartum HIV Care Engagement in the Southern United States, 1999–2016

**DOI:** 10.1093/ofid/ofz023

**Published:** 2019-01-19

**Authors:** Cassandra Oliver, Peter F Rebeiro, Mary J Hopkins, Beverly Byram, Lavenia Carpenter, Kate Clouse, Jessica L Castilho, William Rogers, Megan Turner, Sally S Bebawy, April C Pettit

**Affiliations:** 1 Division of Epidemiology, Vanderbilt University Medical Center, Nashville, Tennessee; 2 Division of Infectious Diseases, Department of Medicine, Vanderbilt University Medical Center, Nashville, Tennessee; 3 Department of Obstetrics and Gynecology, Vanderbilt University Medical Center, Nashville, Tennessee; 4 Vanderbilt Institute for Global Health, Vanderbilt University Medical Center, Nashville, Tennessee; 5 Covance, Clinical Trials, Indianapolis, Indiana

**Keywords:** engagement in care, HIV, postpartum, retention in care, viral suppression

## Abstract

**Background:**

Retention in care (RIC) and viral suppression (VS) are associated with reduced HIV transmission and mortality. Studies addressing postpartum engagement in HIV care have been limited by small sample size, short follow-up, and a lack of data from the Southeast United States.

**Methods:**

HIV-positive adult women with ≥1 prenatal visit at the Vanderbilt Obstetrics Comprehensive Care Clinic from 1999 to 2015 were included. Poor RIC was defined as not having ≥2 encounters per year, ≥90 days apart; poor VS was a viral load >200 copies/mL. Modified Poisson regression was used to estimate adjusted relative risks (aRRs) of poor postpartum RIC and VS.

**Results:**

Among 248 women over 2070 person-years of follow-up, 37.6% person-years had poor RIC and 50.4% lacked VS. Prenatal substance use was independently associated with poor RIC (aRR, 1.40; 95% confidence interval [CI], 1.08–1.80) and poor VS (aRR, 1.20; 95% CI, 1.04–1.38), and lack of VS at enrollment was associated with poor RIC (aRR, 1.64; 95% CI, 1.15–2.35) and poor VS (aRR, 1.59; 95% CI, 1.30–1.94). Hispanic women were less likely and women with lower educational attainment were more likely to have poor RIC. Women >30 years of age and married women were less likely to have poor VS.

**Conclusions:**

In this population of women in prenatal care at an HIV primary medical home in Tennessee, women with prenatal substance use and a lack of VS at enrollment into prenatal care were at greater risk of poor RIC and lack of VS postpartum. Interventions aimed at improving postpartum engagement in HIV care among these high-risk groups are needed.

Approximately 8700 women living with HIV infection in the United States give birth each year, and with pregnancy comes a unique opportunity to engage women in HIV care [1]. Previous studies among pregnant women living with HIV have focused on the prevention of vertical transmission through treatment during pregnancy, and few studies have addressed the postpartum period. If regular medical care is not continued after delivery, then virologic rebound, viral resistance, and HIV transmission can occur [2]. Within the HIV continuum of care framework, antiretroviral therapy (ART) requires adhering to medical appointments and medication to be effective [3]. In keeping with this framework, the National HIV/AIDS Strategy (NHAS) has set goals for 90% of people diagnosed with HIV infection to be retained in care and 90% of those retained in care to be virally suppressed by 2020 [4].

Studies examining postpartum HIV continuum of care outcomes have been limited by lack of psychosocial variables, lack of data on the outcome of postpartum viral suppression, short follow-up periods, and scant data from the Southern United States (a region that comprises 49% of HIV diagnoses but only 37% of the US population) [5–12]. The primary objective of our study was to quantify and identify factors associated with poor postpartum retention in care and viral suppression among HIV-positive pregnant women attending prenatal care at the Vanderbilt Obstetrics Comprehensive Care Clinic (OC3, Nashville, TN) from 1999 to 2016. We aimed to address the limitations of previous studies by including substance use and mental health data, extending follow-up beyond 2 years, and contributing data from the Southern United States.

## METHODS

### Study Population

We conducted an observational cohort study among HIV-positive women ≥18 years of age with ≥1 Vanderbilt Obstetrics Comprehensive Care Clinic (OC3) visit after March 1, 1999, and who gave birth at Vanderbilt University Medical Center (VUMC) before December 31, 2015. All women in prenatal care at the OC3 delivered at VUMC, except ≤20% who delivered at another local health care facility during 2000–2003. Women who had a spontaneous or elective abortion were excluded. We included additional pregnancies per woman if she had a subsequent pregnancy >1 year after the previous pregnancy, allowing for >1 year of follow-up before the next pregnancy. Additional births were treated independently from the index birth, and the women contributed more than 1 person-year for a single 12-month period. Follow-up began at delivery and continued until censoring due to loss to follow-up (LTFU), death, or the end of the study period on December 30, 2016. Vital status was validated by matching to the National Death Index [13]. This study was approved by the Vanderbilt Institutional Review Board.

### Data Collection and Study Definitions

Sources of data included the OC3 data repository and a systematic review of electronic medical records using a standardized data collection form. Women were defined as LTFU and censored if there was no evidence of a health care provider visit within 12 months of the previous visit and there were no future visits. Poor retention in care was defined as not attending ≥2 HIV provider visits ≥90 days apart within 12 months, and poor viral suppression was defined as a last final viral load during the year of interest of ≥200 copies/mL [4, 14, 15]. Both outcomes were measured over each 12-month period after delivery. Therefore, women could have multiple outcomes depending on the year they delivered and the length of their follow-up after delivery. Women who were LTFU were considered not retained in care for the 12-month period before LTFU. If a viral load was missing during any 12-month interval after delivery, the woman was assumed to have a viral load >200 copies/mL.

To be consistent with Centers for Disease Control and Prevention (CDC) HIV Surveillance Reports, we categorized age as 18–24, 25–29, 30–34, 35–39, and 40 years of age or older at delivery [16]. Year of delivery was modeled as a categorical variable in order to maximize flexibility in modeling the association between year of delivery and the outcomes. Race/ethnicity was categorized as white, black American, black African, Hispanic, and other. Separate categories were included for black American women and black African women because black African women have been shown to differ from black American women with respect to HIV transmission risk factors, retention in HIV care, and viral suppression outcomes [17, 18]. We categorized HIV transmission risk factors as heterosexual contact, injection drug use, or other (perinatal transmission and blood product transfusion). Marital status (married or unmarried at the time of the first OC3 visit), educational level (less than a high school education, at least a high school education/general equivalency diploma, or unknown), and insurance status (Medicaid, Medicare, and Ryan White; private; or unknown) were included as socioeconomic indicators. Clinical covariates included self-reported mental health diagnoses (previous self-reported diagnosis of depression, bipolar disorder, schizophrenia, anxiety, and/or cognitive delay), relative timing of HIV diagnosis (during or before the pregnancy of interest), and lack of viral suppression (≥200 copies/mL) at enrollment. Finally, substance use was measured by mandatory urine toxicology conducted during the prenatal period and was defined as positive if any of the following was identified: amphetamines, cocaine, marijuana, or benzodiazepines or opioids that were not prescribed. Insurance status was time-updated during each 12-month period after delivery; the remaining covariates were measured only at enrollment to the OC3.

### Statistical Analysis

Categorical variables were reported as frequency and proportion. Multiple imputation was used to account for missing insurance status (40.3% of person-years) [19]. Missing educational status accounted for <10% of observations; therefore, missing education level was coded simply as unknown educational status.

The adjusted relative risks (aRRs) and 95% confidence intervals (CIs) of poor retention and viral suppression were estimated using modified Poisson regression [20]. The variables included in the multivariable model were chosen a priori based on a literature review and in consultation with specialists in the care of HIV-positive pregnant women.

The largest subgroup for each variable was used as the reference group to improve the statistical stability of the estimates. Generalized estimating equations (GEEs) accounted for multiple outcomes per woman [21, 22]. We used an exchangeable variance–covariance structure and Pan’s quasi-likelihood information criterion to assess improvements to model fit under different specifications of the variance–covariance structure [23, 24]. We estimated predictive margins for the observed values of poor retention in care and viral suppression. We also conducted a Wald test for trends over the entire study period and both before and after 2008, as ART guidelines newly recommended the continuation of ART after delivery at that time [25].

We identified 3 sensitivity analyses to be conducted a priori. For each sensitivity analysis, we compared those included and excluded using the Fisher exact test and ran the full model for poor retention in care and viral suppression. In our primary analysis, we used a retrospective definition of LTFU in order to utilize all available data. Varying definitions of LTFU in HIV observational studies can lead to different estimates of retention and inferences about exposure–outcome relationships [26]. In the first sensitivity analysis, we used a prospective LTFU definition in which women were censored at their first 12-month gap without a visit, regardless of the presence of later visits in the data set. In the second sensitivity analysis, we excluded any births subsequent to the first to determine if the inclusion of more than 1 pregnancy per woman led to biased results. In the third sensitivity analysis, we only included the first 12 months of follow-up for every birth to account for our lack of time-updated variables.

## RESULTS

### Demographic Characteristics

There were 309 deliveries among 248 eligible women over 2070 total person-years of follow-up. The median follow-up time per woman (interquartile range) was 10 (7–14) years, and the median gestational age during first prenatal visit was 15 (12–22) weeks. During study follow-up, 16 (6.5%) women died; the average yearly mortality rate was 0.8%. The baseline demographic characteristics of the study population from their first birth are in Table 1. Of note, black American women accounted for 50.8% (126) of the population, and 8.1% of women (20) were black African. Prenatal substance use was detected for 29.4% (73) of women; 56.2% (41) used marijuana, 49.3% (36) used cocaine, 17.8% (13) used opioids that were not prescribed, 15.1% (11) used benzodiazepines that were not prescribed, and 5.5% (4) used amphetamines. There was an increase in women enrolled with prenatal use of opioids that were not prescribed in the years 2014–2016 from 0.0% of women in 2014 to 8.0% in 2015 and 13.3% in 2016, respectively. The increase between 2014 and 2016 was not significant using a chi-square test (*P* = .09).

**Table 1.** Baseline Characteristics of the Study Population

Characteristic	Women (%)
Total	248 (100)
Age, y	
18–24	72 (29.0)
25–29	84 (33.9)
30–34	50 (20.2)
35–39	36 (14.5)
≥40	6 (2.4)
Race/ethnicity	
Black American	126 (50.8)
White	75 (30.2)
Hispanic	23 (9.3)
Black African	20 (8.1)
Other	4 (1.6)
HIV risk factor	
Heterosexual contact	213 (85.9)
IDU	28 (11.3)
Other	7 (2.8)
Marital status	
Married	66 (26.6)
Unmarried	182 (73.4)
Educational level	
<12th grade	74 (29.8)
≥GED or high school	170 (68.6)
Unknown	4 (1.6)
Insurance status	
Public insurance	109 (44.0)
Private insurance	39 (15.7)
Unknown	100 (40.3)
Mental health diagnosis	
None disclosed	155 (62.5)
Previous diagnosis	93 (37.5)
Timing of HIV diagnosis	
During pregnancy	105 (42.3)
Before pregnancy	143 (57.7)
Viral suppression at enrollment	
Viral suppression	54 (21.8)
Lack of viral suppression	194 (78.2)
Substance use	
Substance use	73 (29.4)
No substance use	175 (70.6)

Abbreviations: GED, general equivalency diploma; IDU, injection drug use.

### Retention in Care

**Figure 1.**
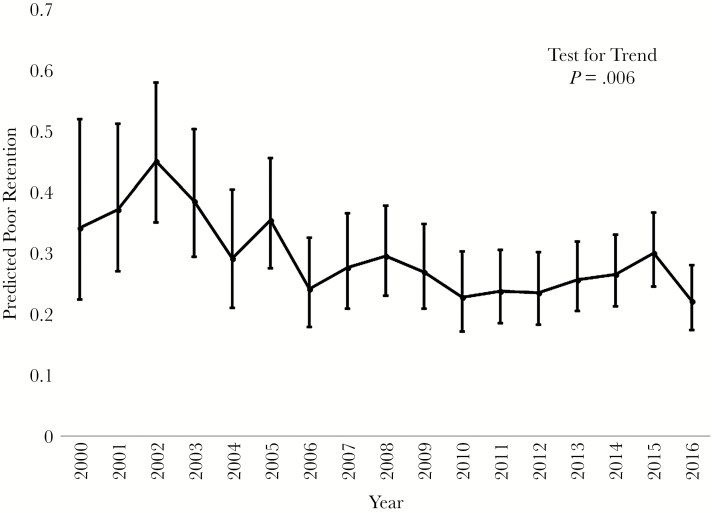
Predicted poor retention in care by year of study. Circles represent point estimates for predicted poor retention in care, and whiskers represent predictive margins for observed poor retention in care after the multivariable regression model.

Overall, 566 (37.6%) person-years (p-y) were not retained in care; 68 (22.0%) p-y were not retained in the first 12 months postpartum. Poor retention in care decreased during the study period from 34% in 2000 to 22% in 2016 (*P* = .006) (Figure 1). Prenatal substance use (aRR, 1.40; 95% CI, 1.08–1.80) and prenatal viral suppression (aRR, 1.64; 95% CI, 1.15–2.35) were independently associated with poor retention. Hispanic women (aRR, 0.56; 95% CI, 0.33–0.95) were less likely to have poor retention in care compared with black American women. Less than a high school education was associated with poor retention as compared with at least a high school education (aRR, 1.29; 95% CI, 1.02–1.64) (Table 2).

**Table 2.** Adjusted and Unadjusted Risks of Poor Retention in Care

Characteristic	Unadjusted Relative Risk (95% CI)	Adjusted Relative Risk^a^ (95% CI)
Age, y		
18–24	1.07 (0.98–1.16)	1.00 (0.76–1.31)
25–29	Ref	Ref
30–34	0.99 (0.90–1.08)	0.89 (0.66–1.21)
35–39	0.94 (0.85–1.03)	0.84 (0.57–1.24)
≥40	0.86 (0.71–1.05)	0.48 (0.23–1.01)
Race/ethnicity		
Black American	Ref	Ref
White	0.95 (0.89–1.03)	0.81 (0.64–1.03)
Hispanic	**0.84 (0.75–0.93)**	**0.56 (0.33–0.95)**
Black African	**0.88 (0.79–0.99)**	0.94 (0.58–1.50)
Other	0.78 (0.53–1.13)	**0.31 (0.11–0.87)**
HIV risk factor		
Heterosexual contact	Ref	Ref
IDU	**1.14 (1.01–1.27)**	1.20 (0.83–1.75)
Other	0.98 (0.82–1.17)	1.03 (0.58–1.85)
Marital status		
Married	**0.91 (0.85–0.97)**	0.90 (0.70–1.15)
Unmarried	Ref	Ref
Educational level		
<12th grade	**1.11 (1.04–1.19)**	**1.29 (1.02–1.64)**
≥GED or high school	Ref	Ref
Unknown	**1.20 (1.25–1.35)**	1.58 (0.96–2.59)
Insurance status		
Public insurance	Ref	Ref
Private insurance	0.99 (0.92–1.06)	1.05 (0.80–1.37)
Mental health diagnosis		
None disclosed	Ref	Ref
Previous diagnosis	1.00 (0.94–1.07)	0.87 (0.69–1.10)
Timing of HIV diagnosis		
During pregnancy	1.03 (0.96–1.10)	0.94 (0.74–1.19)
Before pregnancy	Ref	Ref
Viral suppression at enrollment		
Lack of viral suppression	**1.81 (1.28–2.57)**	**1.64 (1.15–2.35)**
Viral suppression	Ref	Ref
Substance use		
Substance use	**1.17 (1.09–1.26)**	**1.40 (1.08–1.80)**
No substance use	Ref	Ref

Abbreviations: CI, confidence interval; GED, general equivalency diploma; IDU, injection drug use.

^a^Adjusted for all covariates listed in the table, as well as year of delivery.

### Viral Suppression

**Figure 2.**
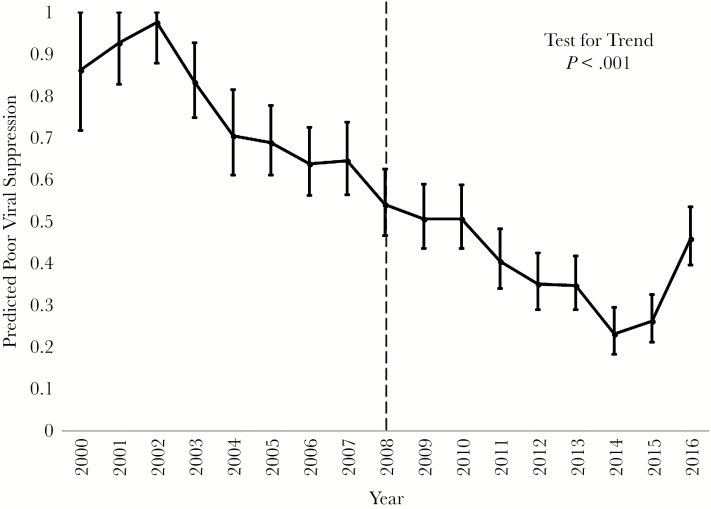
Predicted poor viral suppression by year of study. Circles represent point estimates for predicted poor viral suppression, and whiskers represent the predictive margins for poor viral suppression after the multivariable regression model. In 2008, HIV treatment guidelines changed to recommend continuation of antiretroviral therapy after delivery, as indicated by the dotted line [25].

Overall, 1043 p-y (50.4%) were not virally suppressed postpartum; 169 (54.7%) were not virally suppressed in the first 12 months postpartum. Poor viral suppression decreased from 86% in 2000 to 46% in 2016 (*P* < .001) (Figure 2), but there was a slight increase in 2015 and 2016. Tests for trend in poor viral suppression by year both before and after 2008 were significant (*P* < .001) (Figure 2). Substance use (aRR, 1.20; 95% CI, 1.04–1.38) and prenatal viral suppression (aRR, 1.59; 95% CI, 1.30, 1.94) were associated with poor viral suppression. Older women were less likely to have poor viral suppression (age 30–34 years: aRR, 0.79; 95% CI, 0.67–0.95; age 35–39 years: aRR, 0.69; 95% CI, 0.56–0.85) in comparison with women aged 25–29 years. Married women compared with unmarried women (aRR, 0.84; 95% CI, 0.73–0.96) were also less likely to have poor viral suppression (Table 3).

**Table 3.** Adjusted and Unadjusted Relative Risks of Poor Viral Suppression

Characteristic	Unadjusted Relative Risk (95% CI)	Adjusted Relative Risk^a^ (95% CI)
Age, y		
18–24	**1.17 (1.07–1.28)**	1.02 (0.88–1.17)
25–29	Ref	Ref
30–34	0.92 (0.83–1.01)	**0.79 (0.67–0.95)**
35–39	**0.83 (0.75–0.92)**	**0.69 (0.56–0.85)**
≥40	0.87 (0.71–1.08)	0.84 (0.60–1.19)
Race/ethnicity		
Black American	Ref	Ref
White	1.02 (0.94–1.12)	1.00 (0.89–1.13)
Hispanic	**0.84 (0.74–0.94)**	0.84 (0.65–1.08)
Black African	**0.78 (0.69–0.89)**	0.88 (0.63–1.23)
Other	0.86 (0.56–1.31)	1.05 (0.24–4.57)
HIV risk factor		
Heterosexual contact	Ref	Ref
IDU	**1.18 (1.04–1.35)**	1.15 (0.95–1.40)
Other	0.91 (0.74–1.12)	1.01 (0.75–1.37)
Marital status		
Married	**0.85 (0.79–0.92)**	**0.84 (0.73–0.96)**
Unmarried	Ref	Ref
Educational level		
<12th grade	**1.17 (1.08–1.27)**	1.09 (0.96–1.24)
≥GED or high school	Ref	Ref
Unknown	1.32 (0.98–1.78)	**1.31 (1.07–1.60)**
Insurance status		
Public insurance	Ref	Ref
Private insurance	0.95 (0.87–1.03)	0.93 (0.79–1.09)
Mental health diagnosis		
None disclosed	Ref	Ref
Previous diagnosis	1.01 (0.93–1.09)	1.00 (0.88–1.13)
Timing of HIV diagnosis		
During pregnancy	1.05 (0.97–1.13)	0.94 (0.84–1.05)
Before pregnancy	Ref	Ref
Viral suppression at enrollment		
Lack of viral suppression	**2.09 (1.63–2.68)**	**1.59 (1.30–1.94)**
Viral suppression	Ref	Ref
Substance use		
Substance use	**1.21 (1.12–1.32)**	**1.20 (1.04–1.38)**
No substance use	Ref	Ref

Abbreviations: CI, confidence interval; GED, general equivalency diploma; IDU, injection drug use.

^a^Adjusted for all covariates listed in the table, as well year of delivery.

### Sensitivity Analyses

The alternative prospective LTFU definition decreased the overall p-y contributed by 575 p-y (27.8%). Those contributing to this sensitivity analysis differed from those excluded across person-time based on age, race/ethnicity, HIV risk factor, marital status, insurance status, viral suppression at enrollment, and substance use (Supplementary Table 1). The point estimates for the association of substance use (aRR, 1.29; 95% CI, 0.90–1.86) and viral suppression at enrollment (aRR, 1.46; 95% CI, 0.96–2.22) remained qualitatively the same as in the primary analysis, although they were no longer independently associated with retention in care (Supplementary Table 2). The point estimate for the association of age 30–34 years (aRR, 0.80; 95% CI, 0.64–1.00) with poor viral suppression also remained qualitatively the same as in the primary analysis, although again, it was no longer independently associated with viral suppression (Supplementary Table 3).

By excluding subsequent births, there was a decrease in 61 births (19.7%), accounting for 366 p-y of follow-up (17.7%). The characteristics of the women included in this sensitivity analysis differed from those excluded across person-time for all covariates (Supplementary Table 4). The results of the adjusted model were similar to the results in which subsequent births were included, except that Hispanic ethnicity (aRR, 0.66; 95% CI, 0.39–1.09) was no longer independently associated and being over 40 years of age (aRR, 0.49; 95% CI, 0.29–0.83) was now independently associated with being less likely to have poor retention in care (Supplementary Table 5). The results of the adjusted model for viral suppression were largely unchanged, except that age 30–34 years (aRR, 0.85; 95% CI, 0.70–1.02) was no longer independently associated with poor viral suppression (Supplementary Table 6).

In our third sensitivity analysis, in which we excluded follow-up beyond the first 12 months, there was a decrease of 1761 (85.1%) p-y. The characteristics of the included women within the first 12 months postpartum and those beyond the first 12 months postpartum were similar across person-time except by insurance status and viral suppression at enrollment (Supplementary Table 7). Adjusted regression models were not performed given the loss of >85% of the end points and concern for overfitting the model.

## DISCUSSION

In this population of women in prenatal care, 37.6% of the p-y contributed overall and 22.0% of the p-y contributed in the first year postpartum were not retained in care. This is well below the NHAS goal of only 10% not retained in care by 2020 [4]. However, it is consistent with previous studies’ findings that poor retention ranges from 24% to 71% in the first year postpartum [5–8, 10]. A Southern US study conducted in Jackson, Mississippi, reported that 63% of women were not retained in care in the first year postpartum [8]. It is possible that the markedly higher proportion of women not retained in care in Jackson was due to demographic differences between their study population and ours. For example, in Nashville, Hispanic women were less likely to have poor retention compared with black American women; compared with Nashville, there was a higher proportion of black women (89% vs 58.9%) and a lower proportion of Hispanic women (2% vs 9.3%) included in the Jackson study.

In contrast, the Jackson population had a lower proportion of women using substances during the prenatal period (17% vs 29.4% in Nashville); however, the definition of prenatal substance use likely differed between the 2 studies. In Jackson, the most commonly used substances included cocaine, marijuana, and alcohol; it is unclear if opioid use was assessed. In Nashville, the most commonly used substances included cocaine, marijuana, and opioids; alcohol use was not included in our substance use definition. Opioid use is increasing in the general population and in women during delivery.

In our study, 50.4% of the p-y contributed overall and 54.7% of those contributed during the first 12 months postpartum were not virally suppressed. Other studies that evaluated viral suppression in similar populations found that 56% and 69% lacked viral suppression in the first year after delivery in New York and Philadelphia, respectively [6, 7]. These studies had similar demographic characteristics and used the same definition of viral suppression. Again, this is well below the NHAS goal of <10% without viral suppression by 2020 [4]. The association of younger age, viral suppression at enrollment, and a lack of social support with viral suppression are consistent with previous studies [5, 9, 10]. In a qualitative study, women acknowledged social support as an important factor in HIV care engagment [9]. Studies of the impact of social support interventions (ie, support groups) on postpartum HIV care engagement are warranted.

Lower educational attainment and non-Hispanic ethnicity were associated with poor retention but not with poor viral suppression in our population, and unmarried status was associated with poor viral suppression but not with poor retention in care. Women may differ in characteristics that affect their ability to adhere to medical appointments but not their ability to adhere to medications and vice versa. An example might include stable and reliable transportation, which may impair the ability to adhere to clinic appointments but not to ART. Transportation data were not available in our cohort but should be considered in future studies.

Poor retention in care and viral suppression decreased over the study period (Figures 1 and 2). In 2008, ART guidelines were updated to recommend the continuation of ART after delivery [25]. This guideline recommendation likely contributed, in part, to the observed improvement in poor viral suppression after 2008 [27]. However, poor viral suppression increased during 2015 and 2016. This could possibly be due to the increase in opioid use among pregnant women in recent years [28]. In our study, there was a slight increase from 2014 to 2016 in prenatal opioid use, from 2.6% of women in 2014 to 4.0% in 2016, respectively. Substance use is a modifiable behavior, but studies on the impact of prenatal substance use treatment programs on postpartum HIV care engagement are lacking.

The baseline characteristics of the women included and excluded in the sensitivity analysis, in which women were censored at 1 year postpartum, were similar across person-time with respect to all covariates except for insurance status and viral suppression at enrollment. This is expected given that insurance status is the only variable for which we had time-updated data and women virally suppressed at enrollment were more likely to be retained and followed for a longer period of time. Therefore, in addition to increasing the power of the study, the inclusion of these additional end points likely did not introduce selection bias with respect to baseline covariates. Future studies following women over the long term should incorporate as many time-updated covariates as possible.

This study had several limitations. First, women could have been defined as not retained in care when they transferred care to another clinic. We attempted to investigate the impact of potential misclassification of retention status with our alternative prospective LTFU definition, and our sensitivity analysis showed that there was little impact on our results. Second, our study lacked time-updated data for our baseline covariates. Many of our baseline characteristics (such as substance use) likely change over time, and we were unable to account for this with the data available. Third, systematic and validated data on alcohol use were not available. Lastly, our findings may not be generalizable to the population of pregnant women living with HIV in the United States overall, to those living in other regions of the United States outside of the South, or to women who did not attend prenatal visits.

The strengths of this study include the large sample size and long follow-up period relative to other postpartum studies on engagement in HIV care [5–9]. Additionally, our study population resides in a region of the US disproportionately affected by the HIV epidemic, and the study included the outcome of viral suppression, which has been lacking from previous studies.

## CONCLUSIONS

In conclusion, postpartum HIV care engagement was suboptimal in this Southern US population when compared with NHAS goals. Prenatal substance use, lack of prenatal viral suppression, demographic factors, and socioeconomic factors were associated with poor outcomes. Prospective studies are needed to further explore these relationships with the goal of designing and evaluating an intervention to improve postpartum HIV care engagement.

## Supplementary Data

Supplementary materials are available at *Open Forum Infectious Diseases* online. Consisting of data provided by the authors to benefit the reader, the posted materials are not copyedited and are the sole responsibility of the authors, so questions or comments should be addressed to the corresponding author.

ofz023_suppl_supplementary_material
