# Supplementary material for: Substance Use, Demographic and Socioeconomic Factors Are Independently Associated With Postpartum HIV Care Engagement in the Southern United States, 1999–2016
Source: Open Forum Infect Dis. 2019 Jan 19;6(2):ofz023. doi: 10.1093/ofid/ofz023 (PMC6372056; doi:10.1093/ofid/ofz023)
Supplement: ofz023_suppl_supplementary_material [file ofz023_suppl_supplementary_material.docx]

Supplementary Table 1. Characteristics of Study Population in Person-Years by Excluded and Included in the Alternate Prospective Follow-Up Definition Sensitivity Analysis

| Categories | Person Years Excluded using Alternate Prospective Follow-Up Definition (%) | Person Years Included using Alternate Prospective Follow-Up Definition (%) | p-value* |
| --- | --- | --- | --- |
| Total | 575 | 1495 |  |
| Age |  |  | <0.001 |
| 18-24 | 202 (35.1%) | 362 (24.2%) |  |
| 25-29 | 192 (33.4%) | 520 (34.8%) |  |
| 30-34 | 98 (17.0%) | 321 (21.5%) |  |
| 35-39 | 80 (13.9%) | 252 (16.9%) |  |
| ≥40 | 3 (0.5%) | 40 (2.7%) |  |
| Race/Ethnicity |  |  | <0.001 |
| Black American | 349 (60.7%) | 653 (43.7%) |  |
| White | 166 (28.9%) | 472 (31.6%) |  |
| Hispanic | 23 (4.0%) | 224 (15.0%) |  |
| Black African | 37 (6.4%) | 138 (9.2%) |  |
| Other | 0 (0.0%) | 8 (0.5%) |  |
| HIV Risk Factor |  |  | 0.031 |
| Heterosexual Contact | 528 (91.8%) | 1320 (88.3%) |  |
| IDU | 37 (6.4%) | 120 (8.0%) |  |
| Other | 10 (1.7%) | 55 (3.7%) |  |
| Marital Status |  |  | 0.022 |
| Married | 170 (29.6%) | 521 (34.8%) |  |
| Unmarried | 405 (70.4%) | 974 (65.2%) |  |
| Educational Level |  |  | 0.296 |
| <12^th^ grade | 158 (27.5%) | 432 (28.9%) |  |
| ≥GED or high school | 412 (71.7%) | 1038 (69.4%) |  |
| Unknown | 5 (0.9%) | 25 (1.7%) |  |
| Insurance Status |  |  | 0.036 |
| Public Insurance | 291 (50.6%) | 733 (49.0%) |  |
| Private Insurance | 95 (16.5%) | 320 (21.4%) |  |
| Unknown | 189 (32.9%) | 442 (29.6%) |  |
| Mental Health Diagnosis |  |  | 0.527 |
| None Disclosed | 377 (65.6%) | 958 (64.1%) |  |
| Previous Diagnosis | 198 (34.4%) | 537 (35.9%) |  |
| Timing of HIV Diagnosis |  |  | 0.932 |
| During Pregnancy | 205 (35.7%) | 530 (35.5%) |  |
| Prior to Pregnancy | 370 (64.3%) | 965 (64.5%) |  |
| Viral Suppression at Enrollment |  |  | <0.001 |
| Not Virally Suppressed | 524 (91.1%) | 1126 (75.3%) |  |
| Virally Suppressed | 51 (8.9%) | 369 (24.7%) |  |
|  |  |  |  |
| Substance Use |  |  | <0.001 |
| Substance Use | 172 (29.9%) | 300 (20.1%) |  |
| No Substance Use | 403 (70.1%) | 1195 (79.9%) |  |

*P-value for Fisher's exact test comparing those excluded to those included

Abbreviations: IDU, injection drug use; GED, general equivalency diploma; HIV, human immunodeficiency virus

Supplementary Table 2. Adjusted Relative Risks of Lack of Retention in Care for the Alternative Prospective Follow-Up Definition Sensitivity Analysis

| Characteristic | Adjusted Relative Risk*  Primary Analysis  (95% CI) | Adjusted Relative Risk*  Alternative Prospective Follow-Up Definition  (95% CI) |
| --- | --- | --- |
| Age |  |  |
| 18-24 | 1.00 (0.76,1.31) | 0.95 (0.65,1.39) |
| 25-29 | REF | REF |
| 30-34 | 0.89 (0.66, 1.21) | 1.03 (0.71, 1.50) |
| 35-39 | 0.84 (0.57,1.24) | 0.83 (0.50,1.37) |
| ≥40 | 0.48 (0.23, 1.01) | 0.55 (0.22, 1.32) |
| Race/Ethnicity |  |  |
| Black American | REF | REF |
| White | 0.81 (0.64, 1.03) | 0.79 (0.57, 1.09) |
| Hispanic | **0.56 (0.33, 0.95)** | **0.54 (0.30, 0.95)** |
| Black African | 0.94 (0.58, 1.50) | 0.75 (0.44, 1.28) |
| Other | **0.31 (0.11, 0.87)** | 0.43 (0.18, 1.00) |
| HIV Risk Factor |  |  |
| Heterosexual Contact | REF | REF |
| IDU | 1.20 (0.83, 1.75) | 1.56 (0.99, 2.47) |
| Other | 1.03 (0.58, 1.85) | 1.20 (0.49, 2.94) |
| Marital Status |  |  |
| Married | 0.90 (0.70, 1.15) | 0.97 (0.72, 1.32) |
| Unmarried | REF | REF |
| Educational Level |  |  |
| <12^th^ grade | **1.29 (1.02, 1.64)** | **1.67 (1.21, 2.32)** |
| ≥GED or high school | REF | REF |
| Unknown | 1.58 (0.96, 2.59) | **2.72 (1.37, 5.40)** |
| Insurance Status |  |  |
| Public Insurance | REF | REF |
| Private Insurance | 1.05 (0.80, 1.37) | 1.27 (0.87, 1.86) |
| Mental Health Diagnosis |  |  |
| None Disclosed | REF | REF |
| Previous Diagnosis | 0.87 (0.69, 1.10) | 0.88 (0.65, 1.20) |
| Timing of HIV Diagnosis |  |  |
| During Pregnancy | 0.94 (0.74, 1.19) | 0.91 (0.66, 1.25) |
| Prior to Pregnancy | REF | REF |
| Viral Suppression at Enrollment |  |  |
| Not Virally Suppressed | **1.64 (1.15, 2.35)** | 1.46 (0.96, 2.22) |
| Virally Suppressed | REF | REF |
| Substance Use |  |  |
| Substance Use | **1.40 (1.08, 1.80)** | 1.29 (0.90, 1.86) |
| No Substance Use | REF | REF |

* Adjusted for all covariates listed in the table as well year of delivery

Abbreviations: REF=Referent group; IDU, injection drug use; GED, general equivalency diploma; HIV, human immunodeficiency virus

Supplementary Table 3. Adjusted Relative Risks of Lack of Viral Suppression for the Alternative Prospective Follow-Up Definition Sensitivity Analysis

| Characteristic | Adjusted Relative Risk*  Initial Analysis  (95% CI) | Adjusted Relative Risk*  Alternative Follow-Up  (95% CI) |
| --- | --- | --- |
| Age |  |  |
| 18-24 | 1.02 (0.88, 1.17) | 1.00 (0.83,1.21) |
| 25-29 | REF | REF |
| 30-34 | **0.79 (0.67, 0.95)** | 0.80 (0.64, 1.00) |
| 35-39 | **0.69 (0.56, 0.85)** | **0.67 (0.53, 0.85)** |
| ≥40 | 0.84 (0.60, 1.19) | 0.94 (0.65, 1.37) |
| Race/Ethnicity |  |  |
| Black American | REF | REF |
| White | 1.00 (0.89, 1.13) | 1.00 (0.85, 1.17) |
| Hispanic | 0.84 (0.65, 1.08) | 0.91 (0.68, 1.21) |
| Black African | 0.88 (0.63, 1.23) | 0.91 (0.60, 1.39) |
| Other | 1.05 (0.24, 4.57) | 1.19 (0.26, 5.52) |
| HIV Risk Factor |  |  |
| Heterosexual Contact | REF | REF |
| IDU | 1.15 (0.95, 1.40) | 1.15 (0.92, 1.44) |
| Other | 1.01 (0.75, 1.37) | 0.89 (0.53, 1.51) |
| Marital Status |  |  |
| Married | **0.84 (0.73, 0.96)** | **0.78 (0.65, 0.92)** |
| Unmarried | REF | REF |
| Educational Level |  |  |
| <12^th^ grade | 1.09 (0.96, 1.24) | 1.13 (0.96, 1.32) |
| ≥GED or high school | REF | REF |
| Unknown | **1.31 (1.07, 1.60)** | **1.30 (1.02, 1.65)** |
| Insurance Status |  |  |
| Public Insurance | REF | REF |
| Private Insurance | 0.93 (0.79, 1.09) | 0.95 (0.77, 1.17) |
| Mental Health Diagnosis |  |  |
| None Disclosed | REF | REF |
| Previous Diagnosis | 1.00 (0.88, 1.13) | 1.04 (0.90, 1.22) |
| Timing of HIV Diagnosis |  |  |
| During Pregnancy | 0.94 (0.84, 1.05) | 0.96 (0.83, 1.11) |
| Prior to Pregnancy | REF | REF |
| Viral Suppression at Enrollment |  |  |
| Not Virally Suppressed | **1.59 (1.30, 1.94)** | **1.58 (1.27, 1.96)** |
| Virally Suppressed | REF | REF |
| Substance Use |  |  |
| Substance Use | **1.20 (1.04, 1.38)** | **1.24 (1.02, 1.50)** |
| No Substance Use | REF | REF |

* Adjusted for all covariates listed in the table as well year of delivery

Abbreviations: REF=Referent group; IDU, injection drug use; GED, general equivalency diploma; HIV, human immunodeficiency virus

Supplementary Table 4. Characteristics of Study Population in Person-Years Included in Primary Analysis and Excluded for Subsequent Births

| Categories | Person Years Excluded for Subsequent Births (%) | Person Years Included in Primary Analysis (%) | p-value* |
| --- | --- | --- | --- |
| Total | 366 | 1704 |  |
| Age |  |  | <0.001 |
| 18-24 | 44 (12.0%) | 520 (30.5%) |  |
| 25-29 | 123 (33.6%) | 589 (34.6%) |  |
| 30-34 | 123 (33.6%) | 296 (17.4%) |  |
| 35-39 | 63 (17.2%) | 269 (15.8%) |  |
| ≥40 | 13 (3.6%) | 30 (1.8%) |  |
| Race/Ethnicity |  |  | <0.001 |
| Black American | 106 (29.0%) | 896 (52.6%) |  |
| White | 130 (35.5%) | 508 (29.8%) |  |
| Hispanic | 70 (19.1%) | 177 (10.4%) |  |
| Black African | 60 (16.4%) | 115 (6.7%) |  |
| Other | 0 (0.0%) | 8 (0.5%) |  |
| HIV Risk Factor |  |  | <0.001 |
| Heterosexual Contact | 341 (93.2%) | 1507 (88.4%) |  |
| IDU | 8 (2.2%) | 149 (8.7%) |  |
| Other | 17 (4.6%) | 48 (2.8%) |  |
| Marital Status |  |  | <0.001 |
| Married | 203 (55.5%) | 488 (28.6%) |  |
| Unmarried | 163 (44.5%) | 1216 (71.4%) |  |
| Educational Level |  |  | 0.002 |
| <12th grade | 86 (23.5%) | 504 (29.6%) |  |
| ≥GED or high school | 280 (76.5%) | 1170 (68.7%) |  |
| Unknown | 0 (0.0%) | 30 (1.8%) |  |
| Insurance Status |  |  | <0.001 |
| Public Insurance | 154 (42.1%) | 870 (51.1%) |  |
| Private Insurance | 118 (32.2%) | 297 (17.4%) |  |
| Unknown | 94 (25.7%) | 537 (31.5%) |  |
| Mental Health Diagnosis |  |  | <0.001 |
| None Disclosed | 206 (56.3%) | 1129 (66.3%) |  |
| Previous Diagnosis | 160 (43.7%) | 575 (33.7%) |  |
| Timing of HIV Diagnosis |  |  | <0.001 |
| During Pregnancy | 0 (0.0%) | 735 (43.1%) |  |
| Prior to Pregnancy | 366 (100.0%) | 969 (56.9%) |  |
| Viral Suppression at Enrollment |  |  | <0.001 |
| Not Virally Suppressed | 230 (62.8%) | 1420 (83.3%) |  |
| Virally Suppressed | 136 (37.2%) | 284 (16.7%) |  |
|  |  |  |  |
| Substance Use |  |  | <0.001 |
| Substance Use | 47 (12.8%) | 425 (24.9%) |  |
| No Substance Use | 319 (87.2%) | 1279 (75.1%) |  |

*P-value for Fisher's exact test comparing those excluded to those included

Abbreviations: IDU, injection drug use; GED, general equivalency diploma; HIV, human immunodeficiency virus

Supplementary Table 5. Adjusted Relative Risks of Lack of Retention in Care in which Subsequent Births are Excluded

| Characteristic | Adjusted Relative Risk*  Initial Analysis  (95% CI) | Adjusted Relative Risk*  First Births  (95% CI) |
| --- | --- | --- |
| Age |  |  |
| 18-24 | 1.00 (0.76,1.31) | 1.04 (0.78,1.39) |
| 25-29 | REF | REF |
| 30-34 | 0.89 (0.66, 1.21) | 1.11 (0.80, 1.55) |
| 35-39 | 0.84 (0.57,1.24) | 0.80 (0.51,1.25) |
| ≥40 | 0.48 (0.23, 1.01) | **0.49 (0.29, 0.83)** |
| Race/Ethnicity |  |  |
| Black American | REF | REF |
| White | 0.81 (0.64, 1.03) | 0.86 (0.67, 1.11) |
| Hispanic | **0.56 (0.33, 0.95)** | 0.66 (0.39, 1.09) |
| Black African | 0.94 (0.58, 1.50) | 0.84 (0.48, 1.48) |
| Other | **0.31 (0.11, 0.87)** | **0.31 (0.13, 0.78)** |
| HIV Risk Factor |  |  |
| Heterosexual Contact | REF | REF |
| IDU | 1.20 (0.83, 1.75) | 1.19 (0.80, 1.76) |
| Other | 1.03 (0.58, 1.85) | 1.14 (0.62, 2.10) |
| Marital Status |  |  |
| Married | 0.90 (0.70, 1.15) | 0.87 (0.67, 1.13) |
| Unmarried | REF | REF |
| Educational Level |  |  |
| <12^th^ grade | **1.29 (1.02, 1.64)** | 1.26 (0.98, 1.63) |
| ≥GED or high school | REF | REF |
| Unknown | 1.58 (0.96, 2.59) | 1.42 (0.85, 2.36) |
| Insurance Status |  |  |
| Public Insurance | REF | REF |
| Private Insurance | 1.05 (0.80, 1.37) | 1.01 (0.75, 1.35) |
| Mental Health Diagnosis |  |  |
| None Disclosed | REF | REF |
| Previous Diagnosis | 0.87 (0.69, 1.10) | 0.87 (0.68, 1.11) |
| Timing of HIV Diagnosis |  |  |
| During Pregnancy | 0.94 (0.74, 1.19) | 0.97 (0.75, 1.25) |
| Prior to Pregnancy | REF | REF |
| Viral Suppression at Enrollment |  |  |
| Not Virally Suppressed | **1.64 (1.152.35)** | **1.76 (1.15, 2.70)** |
| Virally Suppressed | REF | REF |
| Substance Use |  |  |
| Substance Use | **1.40 (1.08, 1.80)** | **1.43 (1.10, 1.86)** |
| No Substance Use | REF | REF |

* Adjusted for all covariates listed in the table as well year of delivery

Abbreviations: REF=Referent group; IDU, injection drug use; GED, general equivalency diploma; HIV, human immunodeficiency virus

Supplementary Table 6. Adjusted Relative Risks of Lack of Viral Suppression in which subsequent births are excluded

| Characteristic | Adjusted Relative Risk*  Initial Analysis (95% CI) | Adjusted Relative Risk*  First Births (95% CI) |
| --- | --- | --- |
| Age |  |  |
| 18-24 | 1.02 (0.88, 1.17) | 1.06 (0.92,1.21) |
| 25-29 | REF | REF |
| 30-34 | **0.79 (0.67, 0.95)** | 0.85 (0.70, 1.02) |
| 35-39 | **0.69 (0.56, 0.85)** | **0.73 (0.58, 0.91)** |
| ≥40 | 0.84 (0.60, 1.19) | 1.01 (0.73, 1.41) |
| Race/Ethnicity |  |  |
| Black American | REF | REF |
| White | 1.00 (0.89, 1.13) | 0.97 (0.85, 1.10) |
| Hispanic | 0.84 (0.65, 1.08) | 0.86 (0.67, 1.12) |
| Black African | 0.88 (0.63, 1.23) | 0.68 (0.46, 1.00) |
| Other | 1.05 (0.24, 4.57) | 1.07 (0.25, 4.55) |
| HIV Risk Factor |  |  |
| Heterosexual Contact | REF | REF |
| IDU | 1.15 (0.95, 1.40) | 1.17 (0.96, 1.43) |
| Other | 1.01 (0.75, 1.37) | 1.12 (0.79, 1.58) |
| Marital Status |  |  |
| Married | **0.84 (0.73, 0.96)** | **0.85 (0.73, 0.98)** |
| Unmarried | REF | REF |
| Educational Level |  |  |
| <12^th^ grade | 1.09 (0.96, 1.24) | 1.05 (0.93, 1.20) |
| ≥GED or high school | REF | REF |
| Unknown | **1.31 (1.07, 1.60)** | 1.22 (0.98, 1.53) |
| Insurance Status |  |  |
| Public Insurance | REF | REF |
| Private Insurance | 0.93 (0.79, 1.09) | 0.91 (0.78, 1.07) |
| Mental Health Diagnosis |  |  |
| None Disclosed | REF | REF |
| Previous Diagnosis | 1.00 (0.88, 1.13) | 1.01 (0.89, 1.15) |
| Timing of HIV Diagnosis |  |  |
| During Pregnancy | 0.94 (0.84, 1.05) | 0.98 (0.87, 1.27) |
| Prior to Pregnancy | REF | REF |
| Viral Suppression at Enrollment |  |  |
| Not Virally Suppressed | **1.59 (1.30, 1.94)** | **1.58 (1.26, 1.97)** |
| Virally Suppressed | REF | REF |
| Substance Use |  |  |
| Substance Use | **1.20 (1.04, 1.38)** | **1.21 (1.06, 1.39)** |
| No Substance Use | REF | REF |

* Adjusted for all covariates listed in the table as well year of delivery

Abbreviations: REF=Referent group; IDU, injection drug use; GED, general equivalency diploma; HIV, human immunodeficiency virus

Supplementary Table 7. Characteristics of Study Population in Person-Years by those Included in the Primary Analysis and Exclusion of p-y Beyond 12 months after delivery

| Categories | Person Years Excluded for After 12 Months (%) | Person Years Included for First 12 Months (%) | p-value* |
| --- | --- | --- | --- |
| Total | 1761 | 309 |  |
| Age |  |  | 0.537 |
| 18-24 | 484 (27.5%) | 80 (25.9%) |  |
| 25-29 | 605 (34.4%) | 107 (34.6%) |  |
| 30-34 | 353 (20.0%) | 66 (21.4%) |  |
| 35-39 | 286 (16.2%) | 46 (15.8%) |  |
| ≥40 | 33 (1.9%) | 10 (3.2%) |  |
| Race/Ethnicity |  |  | 0.054 |
| Black American | 854 (48.5%) | 148 (47.9%) |  |
| White | 549 (31.2%) | 89 (28.8%) |  |
| Hispanic | 210 (11.9%) | 37 (12.0%) |  |
| Black African | 144 (8.2%) | 31 (10.0%) |  |
| Other | 4 (0.2%) | 4 (1.3%) |  |
| HIV Risk Factor |  |  | 0.134 |
| Heterosexual Contact | 1581 (89.8%) | 267 (86.4%) |  |
| IDU | 125 (7.1%) | 32 (10.4%) |  |
| Other | 55 (3.1%) | 10 (3.2%) |  |
| Marital Status |  |  | 0.421 |
| Married | 594 (33.7%) | 97 (31.4%) |  |
| Unmarried | 1167 (66.3%) | 212 (68.6%) |  |
| Educational Level |  |  | 0.848 |
| <12th grade | 498 (28.3%) | 92 (29.8%) |  |
| ≥GED or high school | 1237 (70.2%) | 213 (68.9%) |  |
| Unknown | 26 (1.5%) | 4 (1.3%) |  |
| Insurance Status |  |  | 0.009 |
| Public Insurance | 889 (50.5%) | 135 (43.7%) |  |
| Private Insurance | 358 (20.3%) | 57 (18.4%) |  |
| Unknown | 514 (29.2%) | 117 (37.9%) |  |
| Mental Health Diagnosis |  |  | 0.185 |
| None Disclosed | 1146 (65.1%) | 189 (61.2%) |  |
| Previous Diagnosis | 615 (34.9%) | 120 (38.8%) |  |
| Timing of HIV Diagnosis |  |  | 0.632 |
| During Pregnancy | 629 (35.7%) | 106 (34.3%) |  |
| Prior to Pregnancy | 1132 (64.3%) | 203 (65.7%) |  |
| Viral Suppression at Enrollment |  |  | 0.003 |
| Not Virally Suppressed | 1423 (80.8%) | 227 (73.5%) |  |
| Virally Suppressed | 338 (19.2%) | 82 (26.5%) |  |
| Substance Use |  |  | 0.065 |
| Substance Use | 389 (22.1%) | 83 (26.9%) |  |
| No Substance Use | 1372 (77.9%) | 226 (73.1%) |  |

*P-value for Fisher's exact test comparing those excluded to those included

Abbreviations: REF=Referent group; IDU, injection drug use; GED, general equivalency diploma; HIV, human immunodeficiency virus
